# Supplementary figures and images for: Biological factors and statistical limitations prevent detection of most noncanonical proteins by mass spectrometry
Source: PLoS Biol. 2023 Dec 4;21(12):e3002409. doi: 10.1371/journal.pbio.3002409 (PMC10721188; doi:10.1371/journal.pbio.3002409)

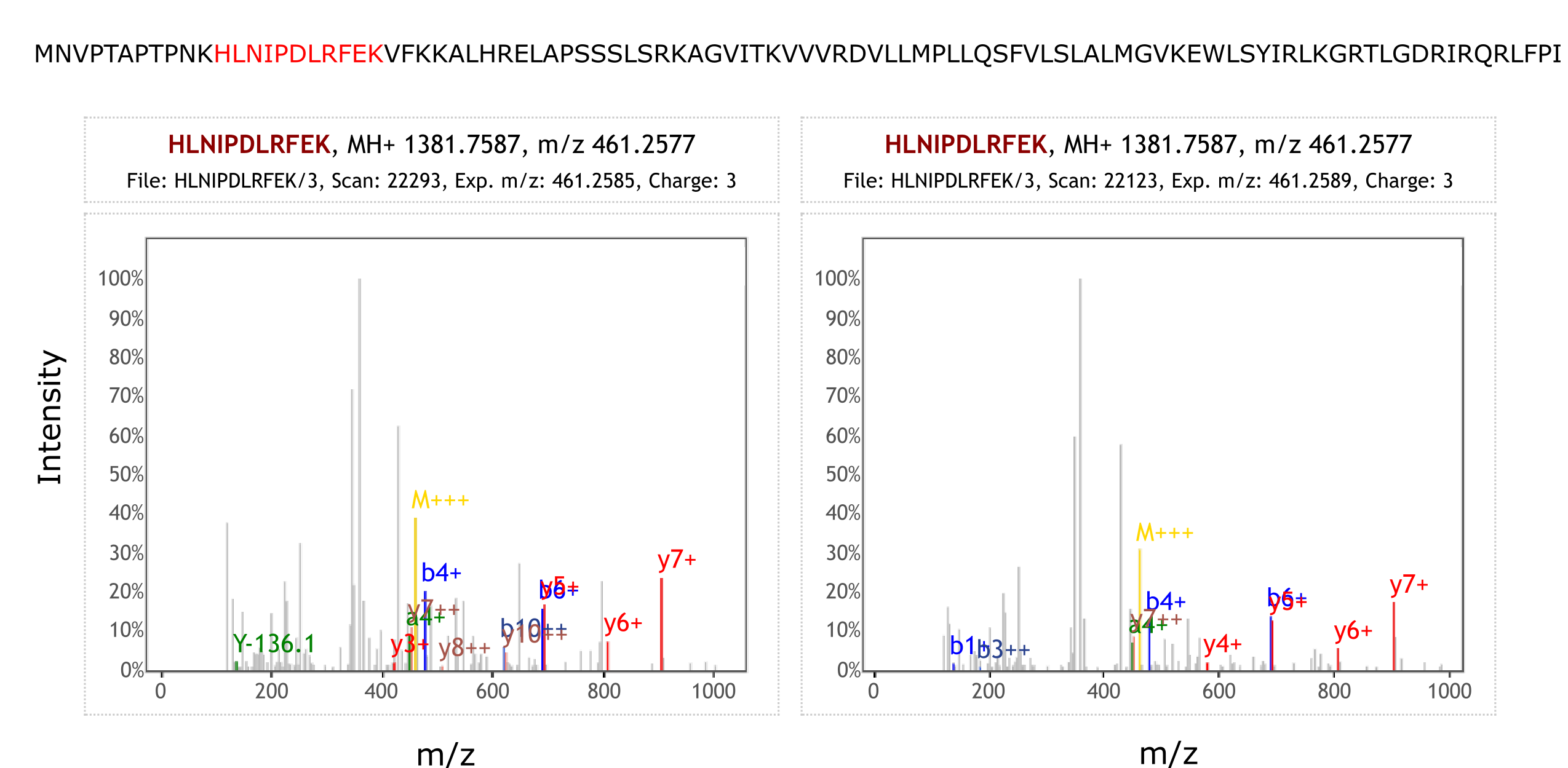

Supplement: S1 Fig — Two spectra with stronger MSFragger expect scores than all decoys are shown. At top, the full protein sequence is shown, with peptide coverage colored in red. Spectra were visualized using the Universal Spectrum Identifier (USI) tool on ProteomeExchange [73] for the USIs given in S1 Table. The data underlying this Figure can be found in S1 Data. (PNG) [file pbio.3002409.s001.png]

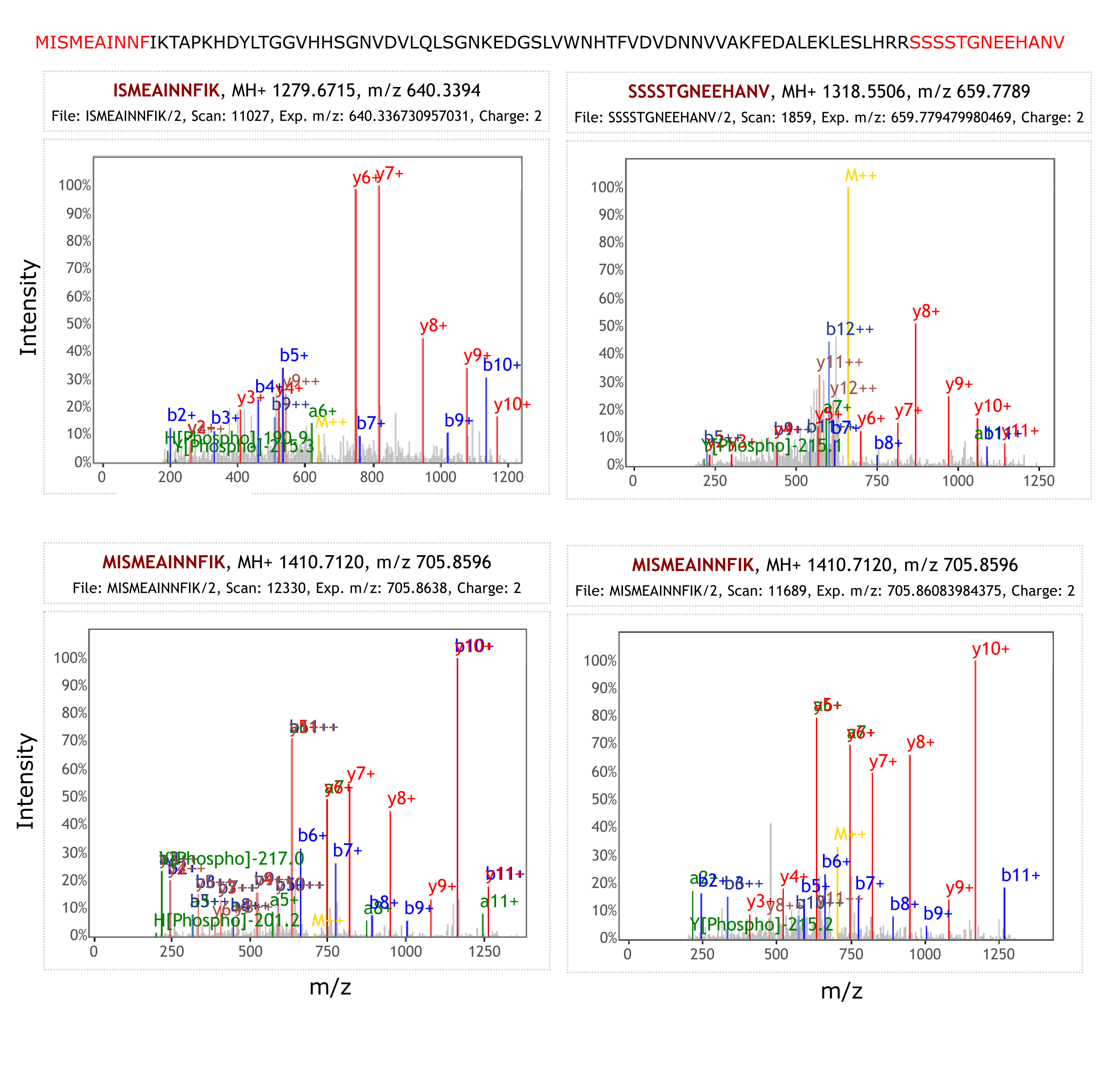

Supplement: S2 Fig — A single PSM (top left) had a stronger MSFragger expect score than all decoys, while 3 others had stronger scores than the next strongest decoy. At top, the full protein sequence is shown, with peptide coverage colored in red. Spectra were visualized using the Universal Spectrum Identifier (USI) tool on ProteomeExchange for the USIs given in S1 Table. The data underlying this Figure can be found in S1 Data. (PNG) [file pbio.3002409.s002.png]

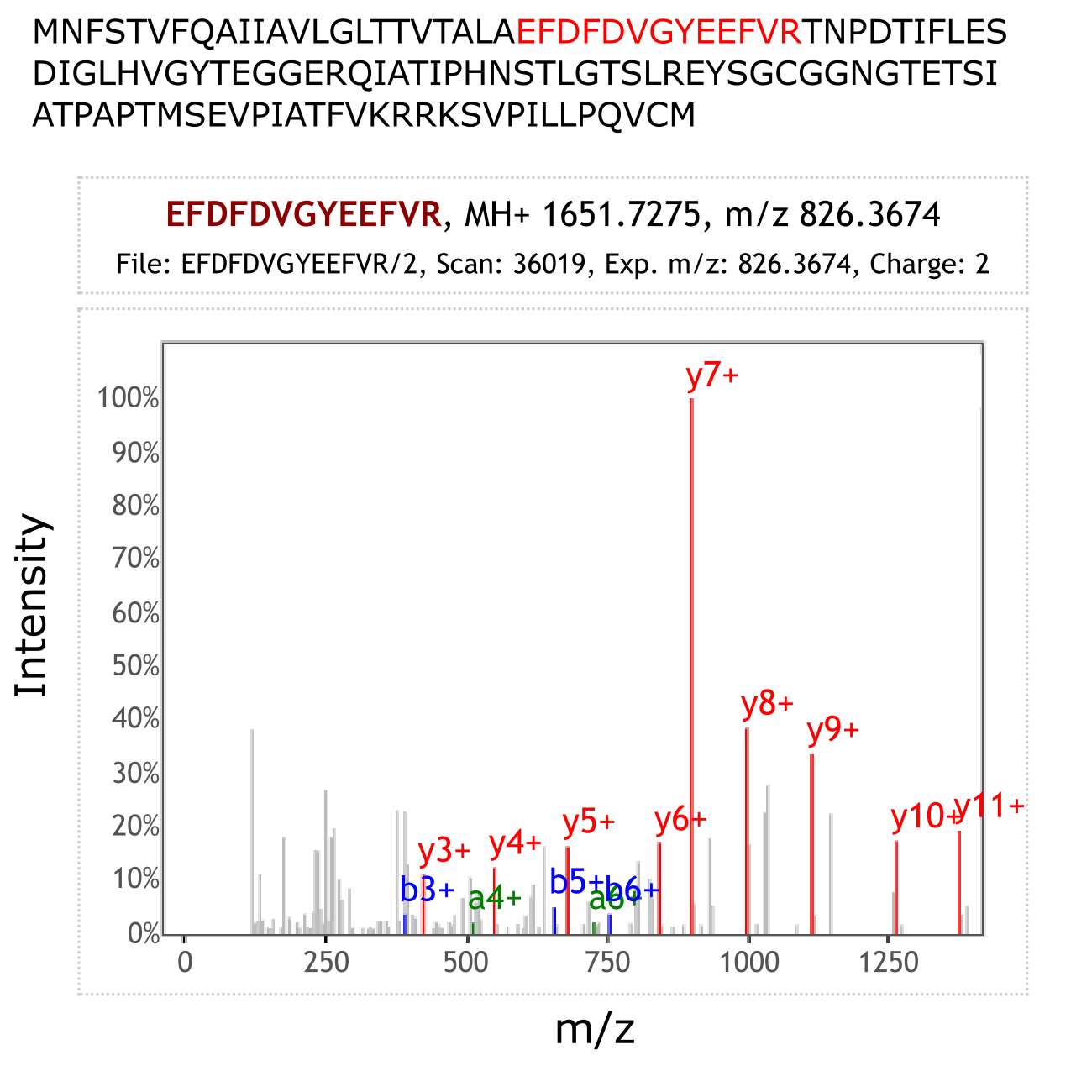

Supplement: S3 Fig — A single PSM had a stronger MSFragger expect score than all decoys when a single non-enzymatic end was allowed. At top, the full protein sequence is shown, with peptide coverage colored in red. The spectrum was visualized using the Universal Spectrum Identifier (USI) tool on ProteomeExchange for the USI given in S1 Table. The data underlying this Figure can be found in S1 Data. (PNG) [file pbio.3002409.s003.png]

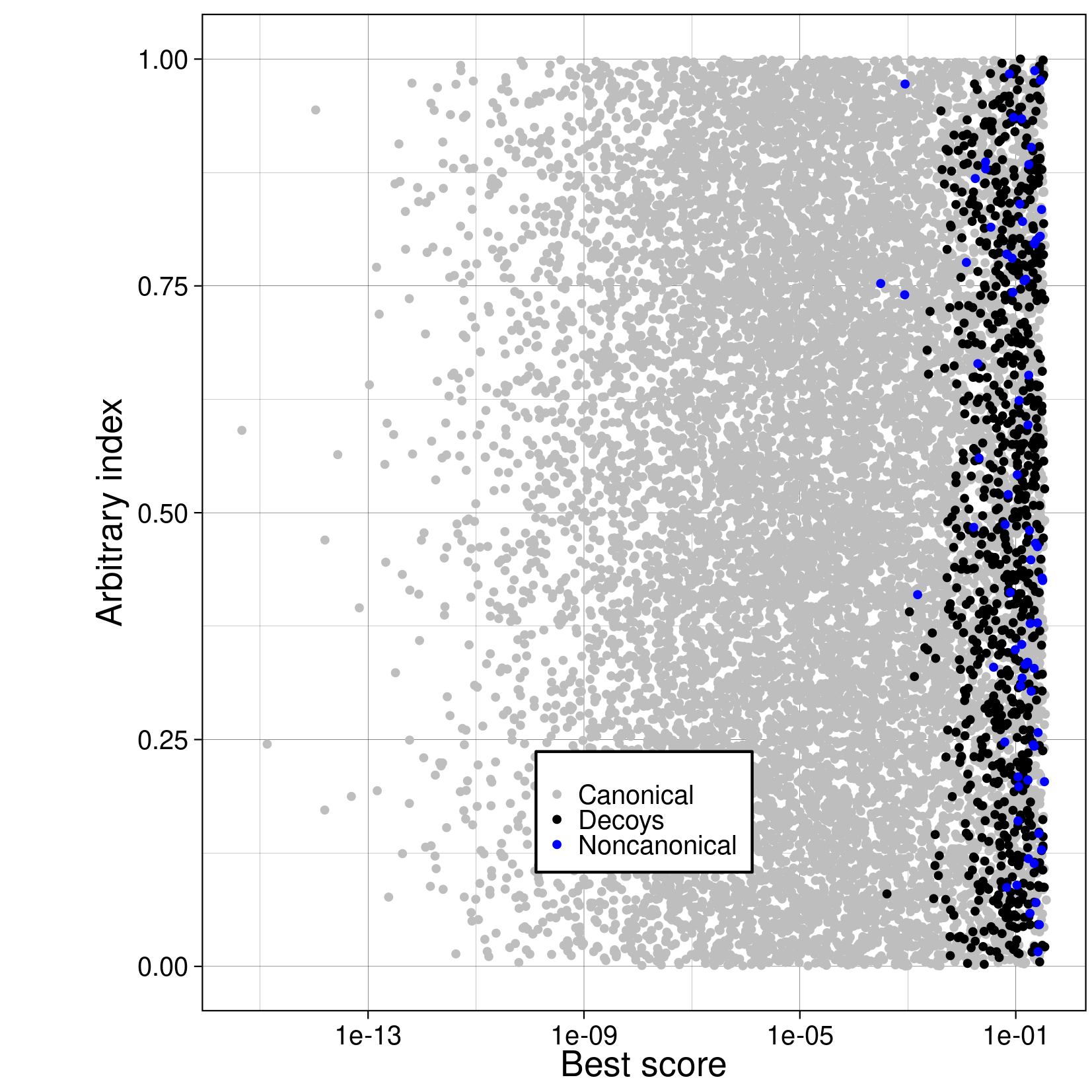

Supplement: S4 Fig — For each protein and decoy passing the detection threshold in the Sun and colleagues [31] study, the strongest score among all PSMs associated with the protein or decoy is indicated. All scores and protein classifications were taken from output files of Sun and colleagues [31] downloaded from IRPOX (PXD028623); we combined all PSMs from 11 different output files to create the plot. Each output file contains the PSMs passing a 1% FDR threshold, set at the whole-proteome level (i.e., not distinguishing canonical from noncanonical), in analyses conducted by Sun and colleagues using pFind. Each output file was individually thresholded at a 1% FDR in the Sun and colleagues’ analysis and noncanonical proteins passing this threshold in any file were inferred to be detected. Lower scores indicate higher confidence given by the MS algorithm. The observation that decoys and claimed noncanonical detections have similar scores suggests that many claimed noncanonical detections (indicated in blue) may be false positives. Merging multiple lists of inferred detections that were each individually generated at a 1% FDR is expected to result in a combined list with a much higher FDR [26], which, together with the use of a proteome-wide rather than noncanonical-specific FDR, can help explain why many noncanonical proteins were inferred to be detected despite scoring similarly to decoys. The data underlying this Figure can be found in S1 Data. (PNG) [file pbio.3002409.s004.png]

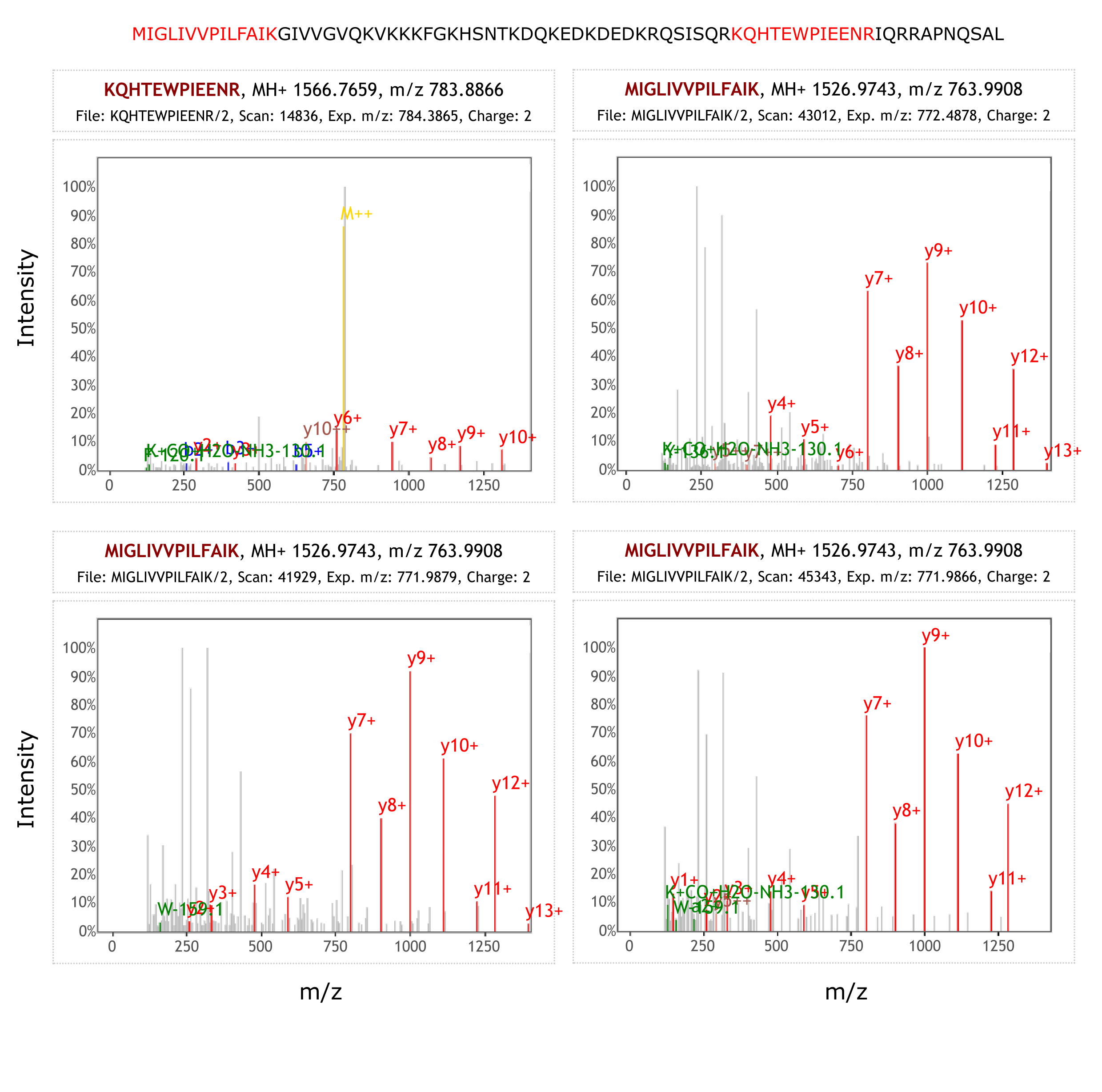

Supplement: S5 Fig — Spectra for the 4 strongest matches for YNL155C-A. At top, the full protein sequence is shown, with peptide coverage colored in red. The spectra were visualized using the Universal Spectrum Identifier (USI) tool on ProteomeExchange for the USIs given in S1 Table. The data underlying this Figure can be found in S1 Data. (PNG) [file pbio.3002409.s005.png]

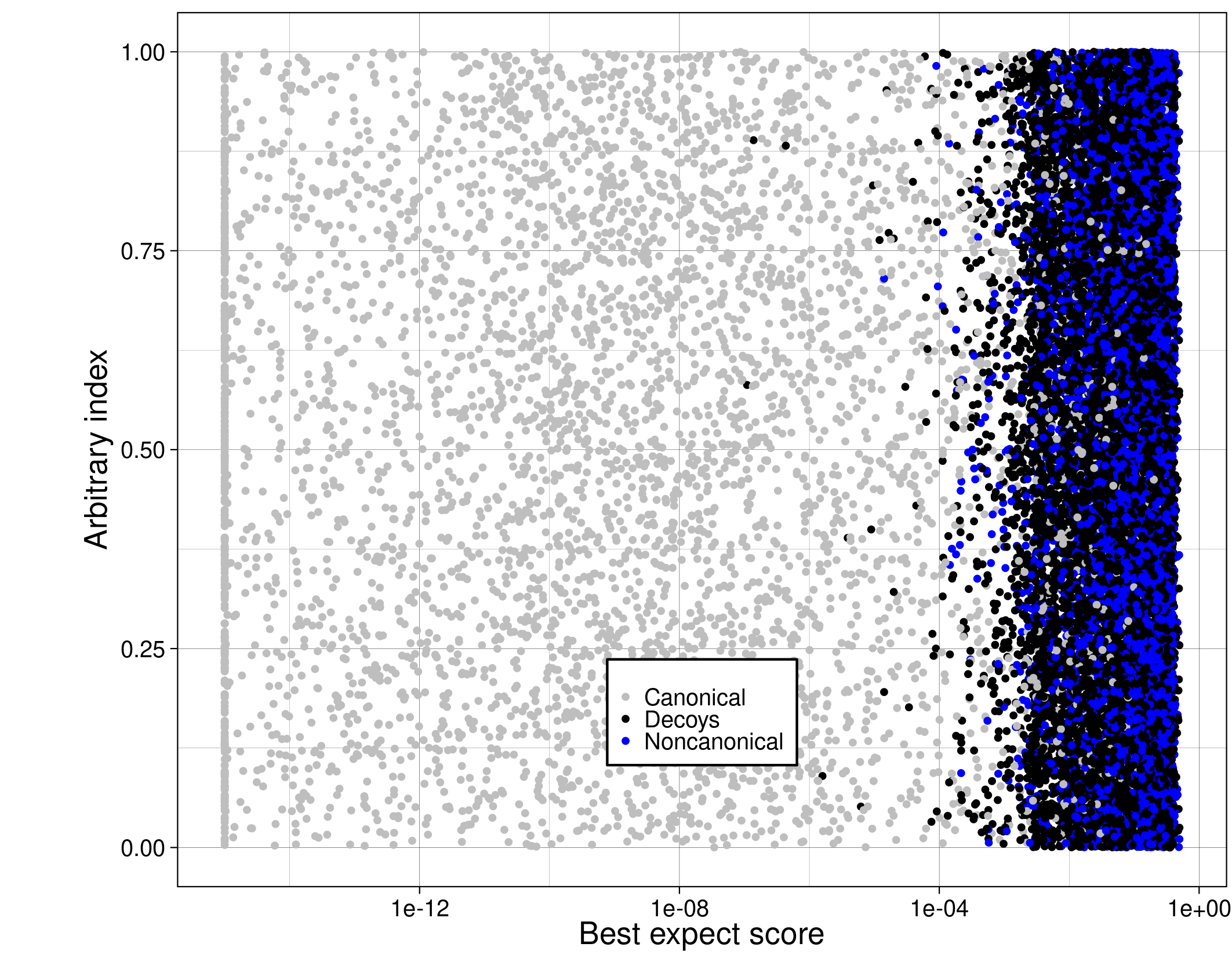

Supplement: S6 Fig — The best peptide-spectrum match MSFragger expect score for each noncanonical protein and decoy in the database. Lower scores indicate stronger matches. The data underlying this Figure can be found in S1 Data. (PNG) [file pbio.3002409.s006.png]

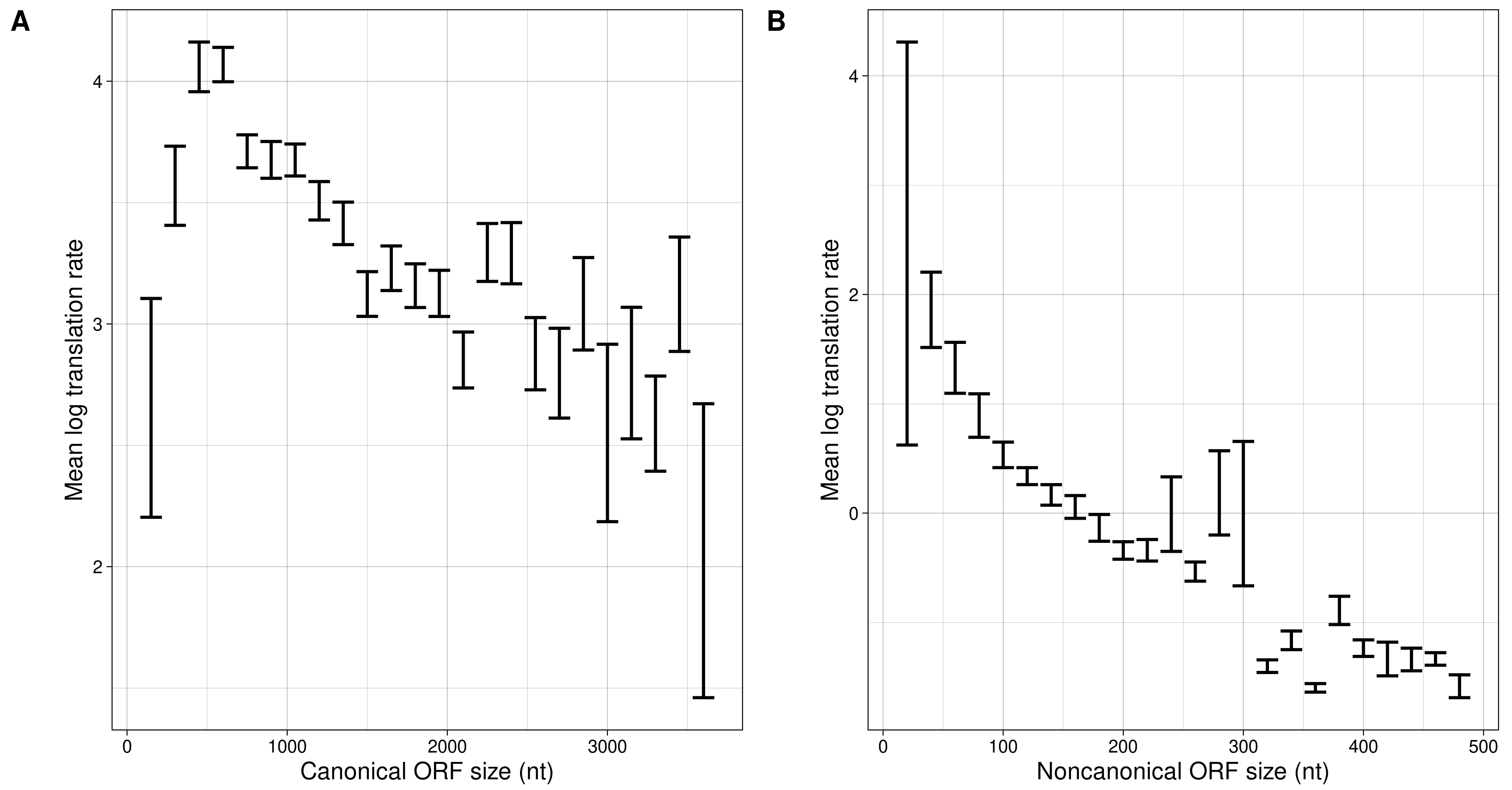

Supplement: S7 Fig — (A) Average log in-frame ribo-seq read count per codon among canonical ORFs of different size classes. (B) Average log in-frame ribo-seq read count per codon among noncanonical ORFs of different size classes. The data underlying this Figure can be found in S1 Data. (PNG) [file pbio.3002409.s007.png]

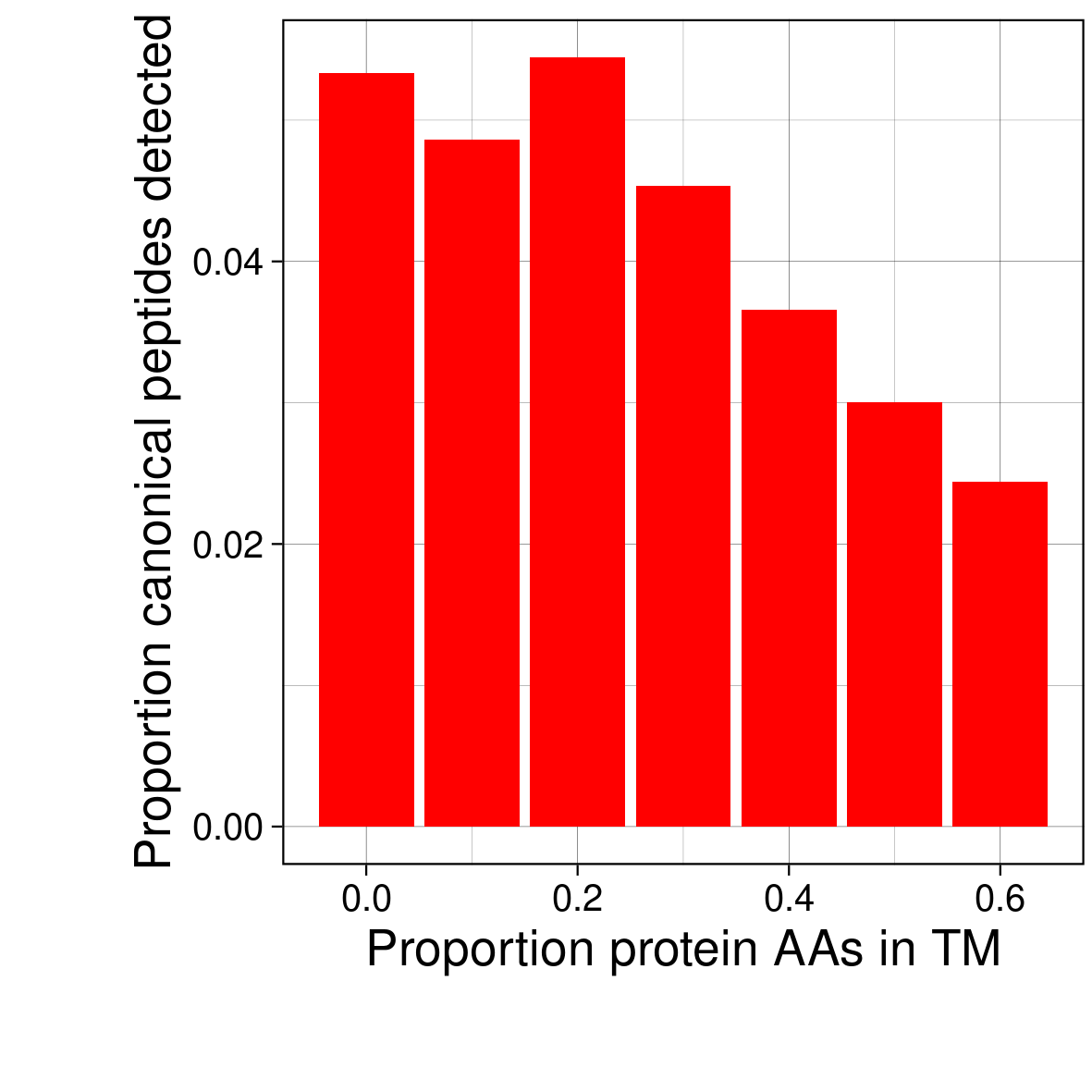

Supplement: S8 Fig — The proportion of peptides detected, among all eligible for detection, for canonical proteins binned by proportion of amino acids in predicted transmembrane domains. Predictions were made using TMHMM [54]. The first bin includes only proteins with no transmembrane domain predicted. The data underlying this Figure can be found in S1 Data. (PNG) [file pbio.3002409.s008.png]

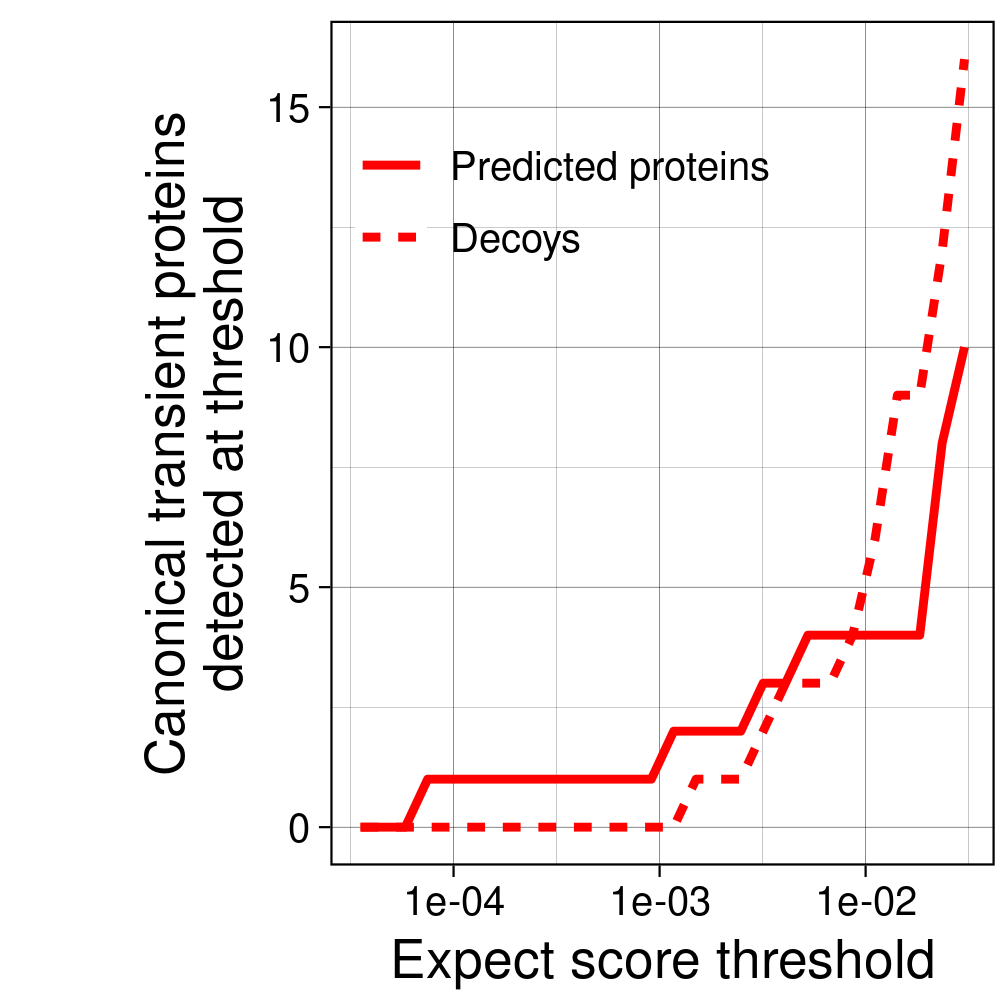

Supplement: S9 Fig — Predicted proteins and decoys detected in MS data at a range of expect-score thresholds, among canonical proteins identified as evolutionarily transient by Wacholder and colleagues [7], using the standard MSFragger approach. The data underlying this Figure can be found in S1 Data. (PNG) [file pbio.3002409.s009.png]
